# Evaluation of Low-Coverage Sequencing Strategies for Whole-Genome Imputation in Pacific Abalone *Haliotis discus hannai*

**DOI:** 10.3390/ijms26104598

**Published:** 2025-05-11

**Authors:** Chengxia Fei, Shoudu Zhang, Xiangrui Chen, Junyu Liu, Wenzhu Peng, Guofan Zhang, Weiwei You, Fucun Wu

**Affiliations:** 1 School of Marine Sciences, Ningbo University, Ningbo 315211, China; ouhaifeichengxia@163.com (C.F.); xiangruichen@126.com (X.C.); 2 CAS and Shandong Province Key Laboratory of Experimental Marine Biology, Center for Ocean Mega-Science, Institute of Oceanology, Chinese Academy of Sciences, Qingdao 266000, China; shouduzhang@163.com (S.Z.); gfzhang@qdio.ac.cn (G.Z.); 3 Marine Science Research Institute of Shandong Province (National Oceangraphic Center, Qingdao), Qingdao 266104, China; 4 State Key Laboratory of Marine Environmental Science, College of Ocean and Earth Sciences, Xiamen University, Xiamen 361102, China; liujy@ysfri.ac.cn (J.L.); pengwenzhu2019@163.com (W.P.); wwyou@xmu.edu.cn (W.Y.); 5 Laboratory for Marine Biology and Biotechnology, Qingdao Marine Science and Technology Center, Qingdao 266000, China; 6 National and Local Joint Engineering Laboratory of Ecological Mariculture, Qingdao 266000, China

**Keywords:** *Haliotis discus hannai*, low-coverage whole-genome sequencing (lcWGS), genotype imputation, accuracy

## Abstract

Low-coverage whole-genome sequencing (lcWGS) followed by imputation is emerging as a cost-effective method for generating a substantial number of single nucleotide polymorphism (SNP) in aquatic species with highly heterozygous and complex genomes. This study represents the first systematic investigation into the application of low-coverage whole-genome sequencing (lcWGS) combined with imputation for genotyping in Pacific abalone (*Haliotis discus hannai*) without a reference panel. We utilized 1059 Pacific abalone individuals sequenced at an average depth of 7.86×, as well as 16 individuals sequenced at 20×, as sample materials. To assess the genotype imputation accuracy for lcWGS without a reference panel, we simulated data with varying sequencing depths (0.5–4×) and examined the effects of sample size, chromosome length, and minor allele frequency (MAF) using BaseVar and STITCH strategies. Results showed that STITCH achieved high accuracy when the sample size exceeded 400, with a genotype correlation (R^2^) of 0.98 ± 0.002 and genotype concordance (GC) of 0.99 ± 0.001. Imputation accuracy plateaued when the sample size exceeded 400 and sequencing depth surpassed 1×. Chromosome length had minimal effects, with all three chromosomes achieving an accuracy of approximately 0.98. However, the accuracy for rare MAF (<0.05) was lower, falling below 0.99. A second imputation with Beagle significantly increased SNP detection by 3.9–8.3 folds for a sequencing depth of 0.5–4×, apparently without sacrificing accuracy. To our knowledge, this is the first study of lcWGS analysis conducted in abalone. The findings demonstrate that lcWGS with imputation can achieve high accuracy with moderate sample sizes (*n* ≥ 400) in Pacific abalone, offering a cost-effective approach for genotyping in aquaculture species.

## 1. Introduction

The Pacific abalone (*Haliotis discus hannai*) is a commercially and ecologically significant species distributed along the coasts of China, Japan, and Korea. It plays a vital role in marine ecosystems by providing abundant food resources for humans and contributing significantly to the ecological balance of ocean environments [1]. In addition to its ecological importance, *H. discus hannai* is a highly valued aquaculture species in East Asia. In China, the annual production of farmed abalone and its hybrids exceeds 200,000 metric tons, making it a major contributor to the aquaculture industry [2]. The cultivation of this species has become a critical livelihood for coastal residents in China, particularly for small- and medium-scale farmers. Likewise, abalone farming in Korea has seen rapid development in recent years, positioning the country as the second-largest producer globally. Furthermore, *H. discus hannai* has been introduced into several European and American countries for aquaculture purposes, further underscoring its global economic significance [3].

In recent years, the rapid growth of the aquaculture industries, combined with advances in high-throughput sequencing technologies, has propelled Pacific abalone research into the genomic era [4]. The evolution of genotyping technologies from low-throughput gel electrophoresis methods to widely used high-throughput approaches has revolutionized the field. High-throughput sequencing technologies are particularly effective in addressing challenges such as high heterozygosity, repetitive sequences, and complex genomic structures [5]. These technologies enable the rapid and comprehensive acquisition of genomic variation data at both the individual and population levels [6], facilitating a deeper understanding of genetic diversity, gene function, and the mechanisms underlying complex traits in aquaculture species [7]. While high-coverage whole-genome sequencing (WGS) remains the gold standard for genotyping, its high cost limits large-scale applications in aquaculture. To overcome this challenge, several cost-effective genotyping technologies have been explored, including SNP chip genotyping [8,9] and reduced representation genome sequencing [10,11]. While SNP chips offer advantages such as lower cost, fast processing, and high data quality, they are inherently limited by their fixed content, which prevents comprehensive coverage of all genetic variants and trait-associated genomic regions [12]. This limitation can compromise the accuracy and completeness of downstream analyses [13].

Low-coverage whole-genome sequencing (lcWGS) has revolutionized genomic studies by offering a cost-effective genotyping method, heralding a new era in genomic studies. Typically performed at ≤1× sequencing depth [14], this technological advancement enables large-scale genetic variant detection through computational imputation, where sparse sequencing data are statistically reconstructed using high-coverage reference genomes [15]. Notably, lcWGS outperforms SNP microarrays in capturing comprehensive variation profiles, particularly in non-coding and structural genomic regions [16]. Although low sequencing depths inherently increase genotype uncertainty, sequencing a large number of individuals can compensate for this by capturing comprehensive population-level genetic diversity [17]. Given the substantial number of missing data points inherent in low-coverage sequencing, genotype imputation plays a pivotal role in integrating population-level information for subsequent analyses [18,19]. Several genotype imputation methods have been proposed in the literature, including Beagle software [20], which uses a Monte Carlo Markov chain (MCMC) algorithm and is widely applied for imputation [21]. In contrast, the STITCH algorithm [8] is notable for its ability to infer population-level ancestral haplotypes from shared haplotype data across a large number of samples. This feature makes it particularly suitable for non-model or non-human species, such as aquatic organisms, where reliable reference panels are often unavailable. STITCH has demonstrated robust performance even at ultra-low sequencing depths [22]. For aquatic species, which often lack large reference populations for functional gene mapping and genomic breeding, STITCH represents a promising approach. However, to date, there have been no published reports on its application in abalone species.

Low-coverage whole-genome sequencing (lcWGS) has proven to be a highly accurate and cost-effective approach for whole-genome SNP genotyping, genomic prediction, and genome-wide association analysis [22,23,24]. Significant progress has been made in its application to livestock and poultry [25,26], and lcWGS has also shown promising potential in aquaculture species such as yellow croaker [27], corals [28], Pacific oysters [29], scallops [30], and salmonid [31]. However, critical barriers persist in adapting lcWGS to marine shellfish like *Haliotis discus hannai*, a species characterized by extreme heterozygosity, fragmented genomes, and absent reference panels. In a study on the yellow croaker, a sequencing depth of just 0.5× across more than 500 individuals achieved an imputation accuracy comparable to that of 8× sequencing [27]. In addition, in another study where low-coverage whole-genome sequencing (lcWGS) was used for genotype imputation in rainbow trout, the concordance obtained was 99.1% [32]. Nonetheless, the application of lcWGS in aquaculture remains in its infancy [33], largely due to the high genetic diversity and recombination rates common in aquatic species. For example, a study on 360 Pacific oyster individuals using lcWGS at an average sequencing depth of 2.82× achieved a genotype accuracy of only 0.860 ± 0.055 [29]. Moreover, aside from the Pacific oyster study, the lack of large-scale reference panels in molluscan species such as Pacific abalone presents additional challenges for genetic inference using lcWGS. This limitation is particularly critical, given the species’ pivotal role in global aquaculture, where rapid genetic improvement is urgently needed to address the environmental stressors and disease outbreaks threatening production sustainability. Currently, optimal genotyping strategies without reference panels have not been established for this species. Therefore, there is an urgent need to develop a systematic and efficient lcWGS-based workflow tailored to the genomic characteristics of aquatic species. Our study bridges this gap by establishing the first methodological framework tailored for high-heterozygosity aquatic species, offering a scalable solution to empower genomic-driven breeding programs in resource-limited aquaculture systems.

In this study, we explored whether low-coverage whole-genome sequencing (lcWGS) combined with an imputation strategy could achieve high genotyping accuracy in *H. discus hannai* without relying on reference panels. A total of 1075 Pacific abalone individuals were sequenced at an average depth exceeding 7× and used as the source material. Low-coverage WGS datasets with depths ranging from 0.5× to 4× were generated by down-sampling reads from these high-coverage data. We evaluated the performance of the low-coverage whole-genome sequencing (lcWGS) strategy in Pacific abalone and investigated the factors affecting genotype imputation accuracy across various sequencing depths, sample sizes, chromosome lengths, and minimum allele frequencies, in the absence of a reference panel. The study aimed to explore the optimal strategies for cost-effective genotype imputation in highly heterozygous marine species. Our findings demonstrate that the BaseVar+STITCH method, followed by secondary imputation with Beagle, performs well and is a robust approach for genotype imputation in low-coverage sequencing data. This research provides valuable recommendations for future whole-genome SNP genotyping in Pacific abalone, with implications for genomic selection, functional gene studies, and other related areas of research in this species.

## 2. Results

### 2.1. SNP Genotyping

**Figure 1:**
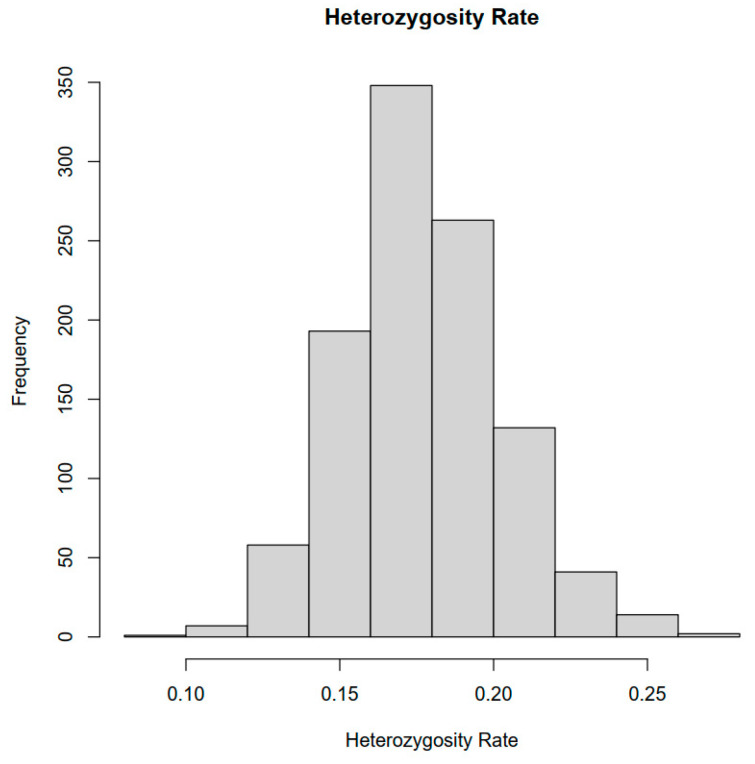
Genome-wide heterozygosity distribution of 1075 Pacific abalone (*Haliotis discus hannai*) samples based on whole-genome sequencing data after quality filtering (average depth 7.86×).

**Figure 2:**
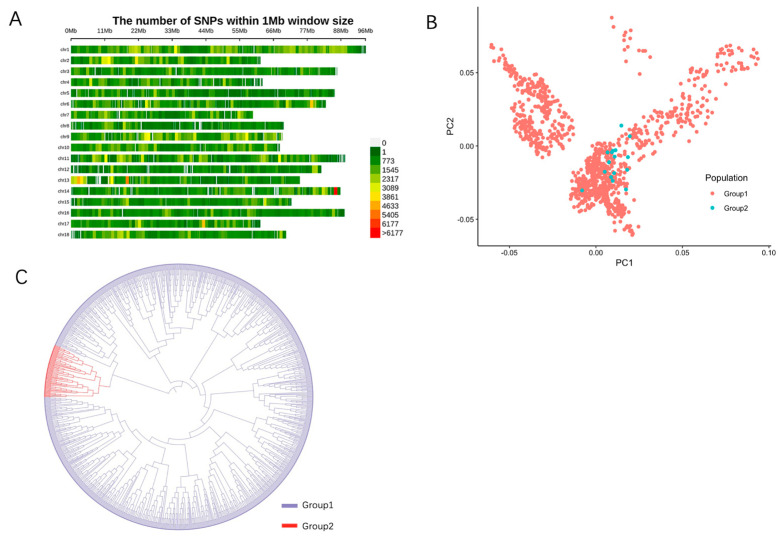
SNP distribution in 1 Mb windows across the genome and genetic structure analysis of the two groups in Pacific abalone. (**A**) Distribution of 1059 Pacific abalone samples’ SNPs in 1 Mb windows across the genome. (**B**) Principal component analysis (PCA) of the first three principal components (Group1, 1059 samples with average coverage depth of 7.86×; Group2, 16 samples with average coverage depth of 20×). (**C**) Phylogenetic tree of samples based on genetic distance (Group1 and Group2 are the same as shown in (**B**)).

After paired-end sequencing and quality control (minor allele frequency, MAF > 0.05), a total of 1479,738 clean reads were generated from 1059 individuals with an average sequencing depth of 7.86×, and 16 individuals with a depth of 20×. The genome quality assessment revealed the Core eukaryotic genes mapping approach (CEGMA) completeness of 85% and benchmarking universal single-copy orthologs (BUSCO) completeness of 93%, with a high small-fragment read pairing rate of 96.95%, and the majority of reads were properly paired. The population genetic structure analysis indicated an average heterozygosity between 0.1 and 0.25 for the 1059 individuals (Figure 1). By calculating Tajima Test (Tajima’s D, positive bias), fixation index (Fst, 0–0.12), and nucleotide diversity (π, 0.001–0.003) for the 1059 individuals (Figure S1), the results showed a rich genetic diversity among these individuals but overall genetic homogeneity. The distribution of 1059 individuals’ SNPs on eighteen chromosomes is shown in Figure 2A; the SNPs across the chromosomes are generally distributed in a relatively uniform manner with an average density of 1260.44 SNPs/Mb. The results show that the distribution of Group1 with 1059 individuals was wide and its internal genetic diversity was high, while the distribution of Group2 with 16 individuals was relatively concentrated, indicating that its genetic homogeneity was strong. However, there were similar genetic backgrounds between the two, which further indicated that there was a genetic exchange between the populations (Figure 2B), and the phylogenetic tree further described the kinship between the two as not extremely close but still moderate (Figure 2C).

### 2.2. Effects of Sequencing Depth and Sample Size on Imputation Accuracy

**Figure 3:**
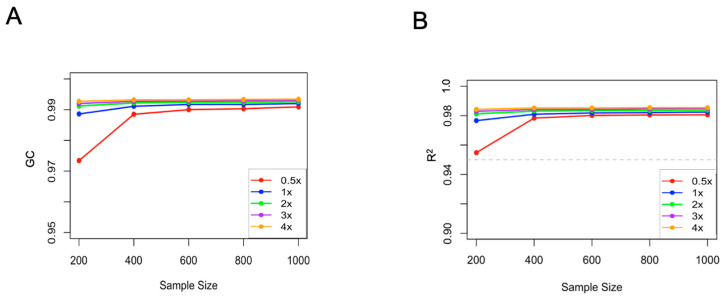
BaseVar+STITCH accuracy of imputation genotype and genotype concordance at different sequencing depths with different population sizes for chromosome 1 in *H. discus hannai*. (**A**) The imputation accuracy of different sequencing depths with different population sizes was measured by genotype concordance (GC). (**B**) The imputation accuracy of different sequencing depths with different population sizes was measured by the squared Pearson correlation coefficient of genotype dosage (R^2^). The dashed line represents the threshold of 0.95.

In our study, the BaseVar+STITCH strategy was employed to evaluate the impact of different sequencing depths and sample size on imputation accuracy. The results indicate that, for varying sample sizes, an increase in sequencing depth from 0.5× to 1× resulted in a 0.1–0.2 improvement in accuracy as measured by the squared Pearson correlation coefficient of genotype dosage (R^2^). With the augmentation of both sample size and sequencing depth, accuracy rose from 0.95 (sample size = 200 and sequencing depth = 0.5×) to 0.99 (sample size = 1075 and sequencing depth = 4×). When the sample size exceeded 400, both genotype imputation accuracy and genotype concordance (GC) content reached a plateau phase (Figure 3). Notably, at a sequencing depth of 0.5× and a sample size of 200, the STITCH imputation accuracy exceeded 0.95. As the sample size surpassed 400 and sequencing depth exceeded 1×, accuracy reached a plateau phase, consistently exceeding 0.98. At a depth of 0.5×, as the sample size increased from 200 to 400, accuracy rose from 0.95 to 0.98. At a depth of 1×, as the sample size increased from 200 to 400, accuracy increased from 0.97 to 0.98. Concurrently, with the increase in sample size and sequencing depth, the GC content also continued to rise, all surpassing 0.97.

### 2.3. Effects of Chromosome Length on Imputation Accuracy

**Figure 4:**
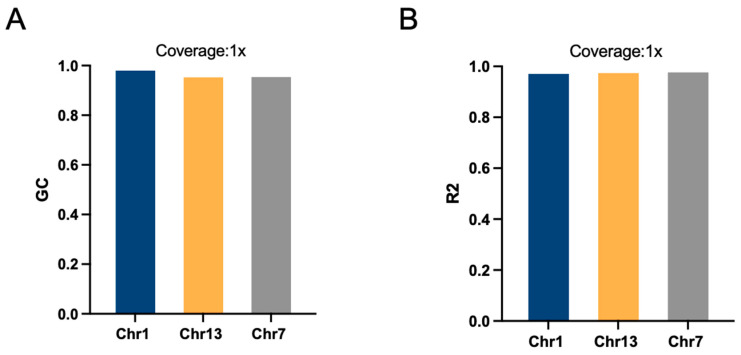
BaseVar+STITCH results of imputation genotype at different chromosome length in *H. discus hannai*. (**A**) The genotype concordance of imputation genotype (GC); (**B**) The squared Pearson correlation coefficient of genotype dosage (R^2^); sample size = 1075, sequencing depth = 1×.

We selected three chromosomes, Chr1, Chr13, and Chr7, of varying lengths, for imputation using 1075 individuals with sequencing depths of 1×, prompting an investigation into the effects of chromosome length on accuracy. The results showed that the imputation accuracy for all three chromosomes exceeded 0.98, with consistency in GC values surpassing 0.97. The accuracy of chromosome 1 was the lowest accuracy, with an R^2^ of 0.982 and GC of 0.992, and that of chromosome 13 was the highest, with an R^2^ of 0.988 and GC of 0.965 (Table 1). The squared Pearson correlation coefficient of genotype dosage (R^2^) and GC did not differ much among the three chromosomes, with Chr 7 having the highest R^2^ and Chr 1 having the highest GC (Figure 4).

### 2.4. Effects of Minor Allele Frequency on Imputation Accuracy

**Figure 5:**
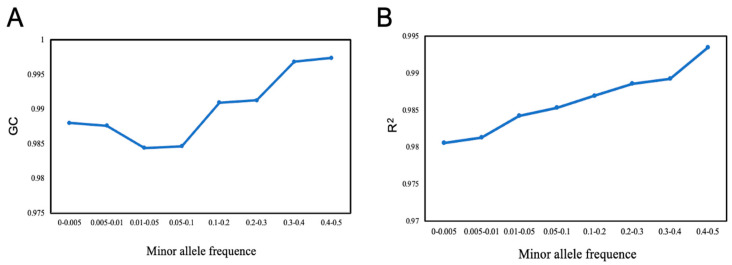
BaseVar+STITCH results of imputation genotype at minor allele frequency (MAF) for chromosome 1 in *H. discus hannai*. (**A**) The genotype concordance of imputation genotype (GC); (**B**) The squared Pearson correlation coefficient of genotype dosage (R^2^); sample size = 1075, sequencing depth = 1×; The SNPs were divided into 8 bins according to their MAF as follows: [0–0.005], [0.005–0.01], [0.01–0.05], [0.05–0.1], [0.1–0.2], [0.2–0.3], [0.3–0.4], and [0.4–0.5].

We used a dataset with sample size of 1075 and a sequencing depth of 1× to evaluate the effect of minor allele frequency (MAF) on accuracy of different methods. The SNPs were divided into eight bins according to their MAF as follows: [0–0.005], [0.005–0.01], [0.01–0.05], [0.05–0.1], [0.1–0.2], [0.2–0.3], [0.3–0.4], and [0.4–0.5]. Figure 5 shows that the effect of MAF was obvious for rare variants with MAF < 0.05; both the genotypic accuracy and the genotypic concordance were greatly affected by MAF; and the accuracy increased rapidly with the increase in MAF. However, MAF > 0.05 had minimal impact on the accuracy of genotype imputation, while genotypic concordance decreased slightly with the increase in MAF.

### 2.5. Evaluation of STITCH and Beagle Strategy

**Figure 6:**
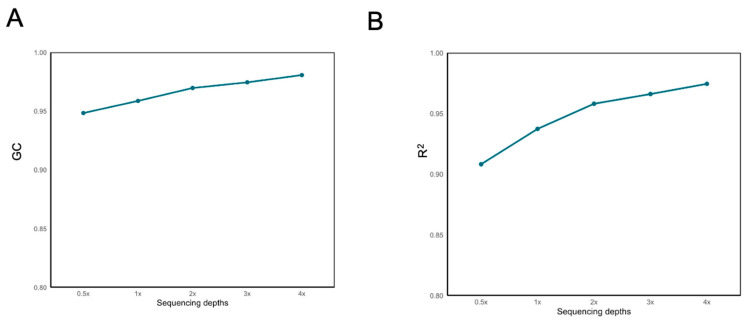
STITCH+BEAGLE strategy results of imputation genotype at different sequencing depths for chromosome 1 in *H. discus hannai*. (**A**) The genotype concordance of imputation genotype (GC); (**B**) The squared Pearson correlation coefficient of genotype dosage (R^2^); sample size = 1075, sequencing depth = 1×.

Due to the persistent high rate of missing data post-STITCH imputation, leading to a reduced count of shared SNPs across individuals, we tried to use the BEAGLE software for a second imputation to the results of STITCH. We assessed the performance of STITCH and BEAGLE with a sample size = 1075 individuals with different sequencing depths. We kept those imputing SNPs in the dataset that were missing rate in ≤20% of the individuals after STITCH imputation and subsequently filled in these missing genotypes using Beagle. With the escalation of sequencing depth and sample size, imputation accuracy exhibited a corresponding increase (Figure 6). As expected, the BEAGLE imputation yielded a substantial rise in the count of SNPs. Table 2 shows the number of SNPs common to all individuals on chromosome 1 after Beagle estimation and their respective estimation accuracies. For missing rate ≤ 20%, compared with the STITCH results, the numbers of SNPs increased from 397.4% (for sequencing depth of 4×) to 646.5% (for sequencing depth of 0.5×), while the imputation accuracies were only slightly reduced. These results proved that the integration of STITCH and Beagle was an effective strategy to imputation and still maintained its high accuracy.

## 3. Discussion

In aquaculture species, the prevailing genotyping strategy for genomic studies has been based on high-coverage sequencing data, typically analyzed using the Genome Analysis Toolkit (GATK) approach. Recently, the introduction of low-coverage whole-genome sequencing (lcWGS) followed by genotype imputation has emerged as a promising, cost-effective alternative for obtaining genome-wide genotypic data in aquaculture [34,35]. The aim of this study was to evaluate the impact of the lcWGS genotyping approach on both the accuracy of SNP imputation and the number of SNPs identified using different strategies in the Pacific abalone *Haliotis discus hannai*. To our knowledge, this is the first study to assess such an approach in Pacific abalone or any other abalone species. Our findings suggest that lcWGS genotyping, coupled with imputation, provides a highly effective method for detecting genome-wide SNP variants in Pacific abalone, offering a robust alternative to traditional high-coverage sequencing for genetic studies in aquaculture. There are various methods for SNP detection, including liquid phase SNP chips [36,37], whole-genome sequencing (WGS) [38], and reduced representation sequencing (RADseq) [39]. However, WGS is prohibitively expensive for large-scale population studies, while reduced representation sequencing and SNP chips have limitations that prevent full coverage of all loci across the genome, making it extremely difficult to identify true quantitative trait loci [40]. This limitation substantially impedes the elucidation of the genetic architecture underlying critical traits and diminishes the reliability of marker-assisted selection in molecular breeding programs.

Missing genotype imputation is a critical component of low-coverage whole-genome sequencing (lcWGS) and plays a pivotal role in its application to aquaculture species [34,41,42]. The effectiveness of the lcWGS genotyping approach is influenced by several factors, including the imputation method, sequencing depth, the number of sequenced individuals (sample size), and the availability and size of a reference panel [43]. In this study, we assessed the imputation performance of lcWGS data in Pacific abalone with respect to these key factors. Specifically, 1075 Pacific abalone individuals, each with sequencing depths exceeding 7×, were used as down-sampling materials for lower coverage depths, while a validation set of 16 individuals with a sequencing depth of 20× was employed to evaluate imputation accuracy. Our findings indicate that high imputation accuracy (>98%) can be achieved in the Pacific abalone population using the BaseVar+STITCH strategy, even in the absence of a reference panel. This represents a significant advancement over the previous aquaculture studies; in *Crassostrea gigas*, lcWGS at a depth of 2.8× achieved only 0.86 accuracy with similar sample sizes [29]. This is particularly relevant, as aquaculture species typically lack large haplotype reference panels and pre-existing variant catalogs [27]. These results are aligned with the previous reports showing that STITCH does not require reference panels for imputation, making it a more suitable approach for aquaculture species [27,28,29]. Moreover, we demonstrate that sequencing depth and sample size significantly affect imputation accuracy. Specifically, a positive correlation was observed between the number of individuals, sequencing depth, and the accuracy of genotype imputation. Notably, an imputation accuracy with and R^2^ exceeding 0.95 was achieved with a sequencing depth of 0.5× and a sample size of 200 individuals. At a sequencing depth of 2×, all sample sizes yielded imputation accuracies surpassing 0.98. However, accuracy tended to plateau beyond a certain threshold. The plateau effect observed at *n* = 400 mirrors the thresholds reported in poultry genomics [25]. These findings are consistent with prior studies in other species, such as Mexican Holstein cattle [44,45], underscoring the critical influence of sequencing depth and sample size on imputation accuracy. Based on our results, we recommend that an imputation accuracy exceeding 98% can be achieved with a sample size of approximately 400 individuals in the Pacific abalone population. Furthermore, sequencing depths between 0.5× and 1× are suggested as optimal for large populations (0.5× for 600 samples and 1× for 400 samples) in the lcWGS approach for Pacific abalone. Above all, we recommend sequencing depths of 1× as optimal for large populations. When the sample size exceeded 400 samples, lcWGS performed very well. This study also highlights the comparable or superior imputation accuracy of the lcWGS approach in aquaculture species, as summarized in Table 3. The impacts of low-coverage depth and sample size on imputation accuracy can be explained by the fact that STITCH uses reads from all BAM files to reconstruct founder haplotypes and perform imputation. As the total number of sequenced individuals and the depth of each sample increase, more reads are available for haplotype reconstruction, leading to a more accurate imputation of the missing whole-genome genotypes [27,46].

The STITCH algorithm, introduced by Davies et al. [8], stands out for its ability to infer genotypes accurately even without a reference panel. It is reported that STITCH had a drawback in that a significant proportion of SNPs remained unimputed even after applying the STITCH imputation method, resulting in the low count of SNPs shared across all individuals as compared to the other methods [25]. In the present study, we conducted a comparative analysis between the STITCH and the STITCH+Beagle imputation strategies. Following a subsequent imputation with Beagle, there was a substantial increase in the number of SNPs, whereas the imputation accuracies were only slightly reduced. This is in accordance with the reports on livestock of Holstein cattle and donkey [25,26]. The present study indicated that STITCH and Beagle were an effective strategy to make up for STITCH’s limitation of reduced SNP yield, while maintaining its advantage of high accuracy [25]. The observed increase in SNP numbers can be attributed to the complementary nature of these imputation methods. STITCH, a reference-free tool, infers missing genotypes from low-coverage sequencing data using population-wide linkage disequilibrium (LD) patterns, but it may struggle with rare variants due to the lack of a reference panel [8]. Beagle, a haplotype-based imputation tool, improves genotype inference by leveraging both LD information and, when available, external reference panels. Its robust algorithm enables accurate imputation across diverse sample types, including low-coverage sequencing data, admixed populations, and pedigrees, demonstrating universal applicability regardless of population structure or sequencing depth [47]. By refining STITCH imputed genotypes with Beagle, additional SNPs can be identified, particularly low-frequency alleles that STITCH alone may miss. Beagle’s superior haplotype reconstruction further enhances SNP detection, explaining the observed SNP increase [25,48]. Using imputation-based sequencing data, the performance of genomic wide association study or evolutionary studies—such as genomic introgression analysis—appears to be strongly influenced by SNP density in genome-wide sequencing [49,50]. For Pacific abalone with no reference panel available, STITCH followed by Beagle would be an optimal strategy to increase the number of SNPs discovered without reducing accuracy.

In this study, we also explored the impact of different chromosome lengths on imputation accuracy. The findings show that the length of the chromosome did not significantly impact the imputation genotype accuracy. This conclusion serves as a guide when discussing the results of imputing low-depth sequencing data on different chromosome lengths using the BaseVar+STITCH strategy. The result was consistent with the previous investigations [51]. In addition, since rare variants are observed only a few times within a population, their imputation is more challenging than that of common variants. This increases the difficulty of establishing haplotype templates while also reducing the available set of matching haplotype references [52]. We delved into the influence of minimum allele frequency (MAF) on imputation accuracy, uncovering a notable decrease in accuracy for MAF < 0.05. This observation parallels previous findings in maize [53] and in sheep [54], highlighting the significance of MAF in imputation accuracy assessments. This finding has important implications for genome-wide association studies (GWAS) and genomic selection (GS), particularly in species like *H. discus hannai*, where rare variants may play crucial roles in key traits such as growth and disease resistance. Due to their low frequency in the population, traditional genotype imputation methods may struggle to accurately infer the true genotypes of these rare variants, potentially impacting subsequent genetic analyses [55]. Compared to the imputation strategy of STITCH, an advantage of another strategy such as GLIMPSE is that it is very robust to MAF. GLIMPSE has been reported to perform quite well (accuracy > 0.9) even for SNPs with an MAF lower than 0.001 [25]. Currently, there are no systematic reports on strategies to improve rare variant imputation using population-specific reference panels. Some studies suggest that selecting loci with a higher MAF can enhance overall imputation accuracy [56]. Alternatively, selecting SNP sites for imputation using an evenly spaced approach can help reduce the genotyping errors caused by low-frequency MAF variants [57].

In aquatic species with highly heterozygous and complex genomes, a coverage of more than 10× should be suitable for obtaining sufficient SNPs with high accuracy [58]. In this study, to assess the imputation accuracies from lcWGS data, we utilized genotyped data from 16 individuals with a sequencing depth of 20× as a validation set. It is important to note that the 1059 Pacific abalone individuals—with a sequencing depth of 7.86×—and 16 individuals—with a sequencing depth of 20×—used for imputation accuracy assessment were able to accurately detect whole-genome SNP variations. From the genomic relationship analysis between the group of 1059 animals and the group of 16 animals in this study, there exists a similar genetic structure but still moderate genetic differentiation between the two populations. This is in accordance with studies where genetic differentiations occurred during a multi-generation selection program in aquaculture [59]. Previous studies in WGS and lcWGS have shown that higher imputation accuracies can be achieved in populations with a close genetic relationship [29]. For instance, in Pacific abalone, improved imputation accuracy is observed in populations with similar genetic structures when using high-depth coverage sequencing data [60]. However, further investigation is needed to determine whether the lcWGS approach is equally effective for multiple populations within the Pacific abalone species. A larger reference panel that provides more comprehensive information about linkage disequilibrium (LD) patterns, as well as stronger LD among SNPs across the genome, can enhance imputation accuracy [27,61]. Such characteristics could contribute to higher imputation accuracy, even across genetically diverse populations.

The findings of this study differ slightly from those reported for other molluscan species, such as the Pacific oyster [29]. In a previous study, genotype imputation using lcWGS data in 300 Pacific oyster individuals at a sequencing depth of 2× achieved an imputation accuracy of GC = 0.951 and R^2^ = 0.890 [29], lower than the imputation accuracy of GC = 0.97 and R^2^ = 0.95 obtained in our study with 200 individuals (sequencing depth of 0.5×). This discrepancy highlights the impact of genomic background on imputation genotype accuracy. It is well documented that the Pacific oyster *Crassostrea gigas* has one of the most complex genomes among molluscan species, characterized by high heterozygosity and genomic variability [62]. As shown in Figure 1, although the Pacific abalone genome is also highly heterozygous and complex, its heterozygosity is approximately 0.15, which is significantly lower than that of the Pacific oyster. Moreover, compared to the relatively low sequencing depth used for validation in the oyster study, our study employed high-depth sequencing (20×) to assess the additional factors influencing imputation accuracy. Our results revealed a strong association between minor allele frequency (MAF) and imputation accuracy. Specifically, imputation accuracy for rare variants was lower, likely due to the significant role that MAF plays in the genetics of complex traits with larger genetic effects [63]. Therefore, enhancing imputation accuracy for rare variants remains an important avenue for future research, as suggested by the present study. Overall, the use of a 20× sequencing depth in this study for accuracy assessment strengthened the reliability of our findings. Additionally, the relatively large sample size enhanced the statistical power of our analyses, improved the imputation performance, facilitated rare variant detection, and provided a more comprehensive understanding of the genetic diversity and population structure in *H. discus hannai*.

This study demonstrates the promising potential of low-coverage whole-genome sequencing (lcWGS) for genomic analysis in Pacific abalone, particularly in the context of cost-effective genomic selection. We evaluated the imputation performance of lcWGS data in relation to sequencing depth and the number of individuals sequenced. Our findings provide valuable insights for both scientific research and applied breeding programs, offering a cost-efficient genotyping strategy that can be integrated into selective breeding pipelines. By optimizing sequencing depth and sample size, hatcheries and breeding programs can enhance genetic evaluations while minimizing costs, ultimately improving production efficiency and sustainability in aquaculture. However, to enhance the reliability and broader applicability of these findings. Future research should focus on developing high-quality reference panels to improve imputation accuracy, particularly for rare variants. Exploring advanced imputation algorithms and hybrid methods could further enhance performance. Increasing sample sizes and optimizing sequencing depth across diverse populations would improve statistical power and cost-effectiveness. Comparative studies on genomic background effects and integrating long-read sequencing or multi-omics approaches could refine imputation strategies. Additionally, haplotype-based methods and functional annotations may enhance rare variant detection. These improvements will strengthen the application of low-coverage whole-genome sequencing in Pacific abalone breeding and genomic studies.

## 4. Materials and Methods

### 4.1. Sample Materials and Whole-Genome Sequencing

A total of 1075 Pacific abalone individuals were randomly selected as samples from a selective breeding program in Fuda Abalone Farm (Jinjiang, China) for whole-genome sequencing, as described by Liu et al. [36]. To ensure population representativeness, individuals were selected from 114 families, including 86 paternal half-sibling families and 16 maternal half-sibling families, with 57 fathers and 106 mothers. In this study, normality tests were conducted using the phenotyping data of the animal samples that were chosen for sequencing. The whole-genome sequencing (WGS) data of 1059 abalone were obtained, with an average sequencing depth of 7.86× (at Novogene Corporation (Beijing, China), using the Illumina NovaSeq 6000 platform (150-bp paired-end; Illumina, Sacramento, CA, USA). In addition, in order to eliminate the potential impact of inaccurate genotyping due to relatively low measurement depth (7.86×), this study used an additional 16 individuals with an average resequencing depth of 20× as validation samples.

### 4.2. Sequencing Analysis and Variant Calling

A total of 1075 individuals were sequenced using the Illumina sequencing platform, with an average depth of 7.86×. The raw data were filtered using fastp (v0.23.4, Opengene, Shenzhen, China) to remove low-quality or adapter sequences [64]. The clean reads were then aligned to the reference genome of abalone (GenBank: GCA_044707095.1) (1.4 Gb) [36] using the mem algorithm of BWA (Burrows–Wheeler Aligner, v0.7.17, Boston, MA, USA) [65], and the resulting BAM files were sorted using SAMtools (v1.18, Boston, MA, USA). The sorted and aligned BAM files were processed with the MarkDuplicates algorithm from GATK (v4.2.2, Broad Institute, Cambridge, MA, USA) to flag duplicates [66], with default parameters. Variants were filtered with the GATK VariantFiltration parameter “QD < 2.0 || MQ < 40.0 || FS > 60.0 || SOR > 3.0 || MQRankSum < 12.5 || ReadPosRankSum < −8.0”. Following hard filtering with GATK, the SNPs were further filtered using VCFtools (v0.1.16, Wellcome Sanger Institute, Hinxton, Cambridgeshire, UK) [67] for subsequent analyses.

To evaluate the genomic relationship between the 16 samples with a sequencing depth of 20× and 1059 samples with an average sequencing depth of 7.86×, imputation of the missing genotypes in the whole-genome sequencing data was performed using Beagle (v5.1, University of Washington, Seattle, WA, USA) [68]. Variants with a minor allele frequency (MAF) lower than 0.05 and a deviation from the Hardy–Weinberg equilibrium (HWE) (*p* value < 10^−7^) were excluded using the PLINK software (v 1.90, Broad Institute, Cambridge, MA, USA) [69]. Furthermore, due to the high level of LD in the genome, most SNPs were redundant. LD pruning was performed using PLINK (v 1.9) [69] to remove variants with high LD (R^2^ > 0.9). After pruning, 1,642,965 SNPs were retained in the whole-genome sequencing dataset. Principal component analysis (PCA) was performed on the genomic relationship matrix using the GCTA software (v1.25.3, Westlake University, Hangzhou, China) [70]. This resulted in a matrix of eigenvectors in descending order that represented principal components (PCs), where PC1 had the largest eigenvalue. The overall structure of genetic variation was visualized using a scatterplot of the top few PCs. Furthermore, we used MEGA (v 11.0.13, Temple University, Philadelphia, PA, USA) [71] to construct a phylogenetic tree to illustrate the genetic relationships.

### 4.3. Genotype Imputation

We used two methods to impute low-depth sequencing data. The Beagle software (v 5.1) [68] was employed as the first method, which leveraged the linkage disequilibrium information between genetic markers to impute the genotypes for missing loci, thereby enhancing the accuracy of the genotype data in the samples. The second method used was the STITCH software (v1.7.0, University of Oxford, Oxford, Oxfordshire, UK) (Sequencing to Imputation Through Constructing Haplotypes, which performed genotype imputation based on a reference haplotype library, eliminating the need for additional reference panels [8]. In this study, the following two strategies were primarily adopted for imputation: BaseVar+STITCH for the initial imputation and Beagle for the secondary imputation. BaseVar (v0.8.0, Beijing Genomics Institute, Shenzhen, Guangdong, China) was primarily employed to infer allele frequencies and identify polymorphic sites, followed by imputation using STITCH. Subsequently, the SNPs with a missing rate of 0.2 were selected for the secondary imputation using Beagle.

#### 4.3.1. Evaluation of the Impact of Different Sequencing Depths and Sample Sizes on Imputation Accuracy

To detect whether and how the accuracy of genotype imputation is influenced by sequencing depth and sample size in Pacific abalone, we used the BaseVar+STITCH strategy to further investigate the relationships. The BaseVar software, developed by BGI, was applied to extract variant position information and infer allele frequencies [72]. In this study, we randomly down-sampled paired-reads from the 1075 sequenced individuals to produce different sequencing datasets with depths of 0.5×, 1×, 2×, 3×, and 4×, respectively, using the DownsampleSam module of the Picard software (v2.9.0, Broad Institute, Cambridge, MA, USA) [73]. Sequencing depths (0.5×, 1×, 2×, 3×, 4×) were chosen based on cost–benefit thresholds observed in previous aquatic studies [29]. Additionally, we sampled 200, 400, 600, and 800 individuals that were randomly selected from the 1075 sequenced samples to generate samples of diminished sizes. The sample sizes were selected to span from the minimum effective population size to the saturation points identified in previous genomic studies [25]. STITCH was then employed to impute the data on chromosome 1, enabling the assessment of imputation accuracy.

#### 4.3.2. Evaluation of the Impact of Chromosome Length on Imputation Accuracy

To evaluate the influence of chromosome length on genotype imputation in Pacific abalone, considering the differences in imputation stability observed across chromosomes [74] and the computational time limitations [75], we selected a sample size of 1075 and three chromosomes—Chr 1, Chr 13, Chr 7—representing the long, medium, and short chromosomes, respectively, to assess the imputation accuracy at a sequencing depth of 1×. The variant position information of Chr 1, Chr 7, and Chr 13 was extracted from all the de-weighted bam files using the BaseVar software (v0.8.0). The three chromosomes were then imputed and assessed for accuracy.

#### 4.3.3. Evaluation of the Effect of Allele Frequency on Imputation Accuracy

The minor allele frequency (MAF) is indicative of the frequency of a specific allele within a population, impacting the genotypes considered and imputed during genotype imputation. Lower MAF values may result in the neglect or erroneous imputation of rare genotypes, while higher MAF values could enhance imputation accuracy but also increase computational time. To assess the impact of MAF on imputation accuracy, the MAF was divided into eight intervals using the vcftools (v0.1.16) software—[0–0.005], [0.005–0.01], [0.01–0.05], [0.05–0.1], [0.1–0.2], [0.2–0.3], [0.3–0.4], and [0.4–0.5]—and the accuracy was compared at a sequencing depth of 1×.

### 4.4. Evaluation of Imputation Accuracy

To assess the accuracy of genotype imputation in the low-coverage whole-genome sequencing (lcWGS) of Pacific abalone, a validation set comprising 16 individuals with an average sequencing depth exceeding 20× was randomly selected. In this study, we considered the use of 16 validation individuals to be sufficient. For example, in the study by Yang et al., 36 individuals with a sequencing depth of 12× were used for validation, while another study used a subset of 18 individuals sequenced at a depth of 30× as validation samples [12,29]. High sequencing depth provides highly accurate genotype information, and the selected individuals share a similar genetic background with the 1059 imputed individuals, making them a reliable “true” genotype reference. The increased sequencing depth significantly reduces sequencing errors and random noise in genotype calls, ensuring the reliability of the validation results. The following two evaluation metrics were employed: genotype concordance (GC) and the squared Pearson correlation coefficient of genotype dosage (R^2^). Genotype concordance (GC) evaluates the consistency between imputed genotypes and those determined through high-depth sequencing, serving as a measure to compare the agreement between the two [76]. The squared Pearson correlation coefficient of genotype dosage (R^2^) is used to quantify the accuracy of imputed genotypes as compared to genotypes obtained from high-depth sequencing. It reflects the squared correlation between the posterior expectation of imputed allele dosages and the true genotypes derived from the high-depth sequencing data; the high-depth data serve as the reference, and low-depth data serve as the test set [54]. Genotyping was performed on the sequencing data of these individuals. Both metrics were calculated for each SNP, and the mean value across all SNPs was determined. This systematic evaluation, utilizing GC and R^2^ metrics, established a robust framework for assessing the accuracy of genotype imputation within the lcWGS context, ensuring a comprehensive and rigorous evaluation of the imputation quality against high-depth sequencing data.

## 5. Conclusions

This study provides a comprehensive evaluation of low-coverage whole-genome sequencing (lcWGS) as a cost-effective and reliable genotyping method for Pacific abalone. The following key conclusions can be drawn from our research: (1) Our results suggest that an imputation accuracy above 98% can be achieved with a sample size of approximately 400 individuals, making lcWGS a robust method for genomic studies in aquaculture species. (2) A sequencing depth of 1× is recommended as the optimal balance between cost and accuracy for large-scale population studies. When the sample size exceeds 400 individuals, lcWGS exhibits outstanding performance in variant detection efficiency. (3) For Pacific abalone lacking a reference panel, the combination of STITCH followed by Beagle represents an optimal strategy to increase the number of SNPs discovered, which is crucial for improving genetic selection in Pacific abalone. (4) These findings establish low-coverage whole-genome sequencing (lcWGS) as a viable and cost-effective genotyping method for Pacific abalone, providing a solid foundation for future analyses in this species and potentially other aquaculture species with complex genomes. Collectively, this study provides valuable insights into the implementation of high-throughput genotyping technologies, which can accelerate the genetic analysis of economic traits in aquaculture species.

## Figures and Tables

**Table 1:** The squared Pearson correlation coefficient of genotype dosage (R^2^), genotype concordance (GC), chromosome length, and SNP density revealed by lcWGS approach among different chromosomes in *H. discus hanna*.

Chr	R^2^	GC	Chromosome Length	SNP Density (bp/SNP)
1	0.982	0.992	96,096,873	96.983
7	0.985	0.966	59,286,736	131.189
13	0.988	0.965	74,588,556	102.116

**Table 2:** Number of SNPs and Genotype imputation accuracy with the squared Pearson correlation coefficient of genotype dosage(R^2^) under different sequencing depths for STITCH and STITCH + Beagle (sample size = 1075, chromosome 1, Missing rate ≤ 0.2).

Sequencing Depth	STITCH		STITCH+BEAGLE	
	No. of SNPs	R^2^	No. of SNPs	R^2^
0.5×	114,056	0.9805	851,527	0.9082
1×	147,449	0.9824	1,369,351	0.9373
2×	210,381	0.9837	1,369,982	0.9580
3×	247,669	0.9848	1,369,982	0.9660
4×	275,461	0.9854	1,370,182	0.9744

**Table 3:** Previous publications on aquaculture animals, with species, sequencing depth, sample size, imputation accuracies, and number of SNPs.

Species	Sequencing Depth	Sample Size	Imputation Accuracy	SNPs Number	Publication
*Crassostrea gigas*	2.8×	≥300	0.860	11,000,000	[29]
*Larimichthys crocea*	0.5×	536	0.795	5,949,426	[27]
*Acipenser gueldenstaedtii*	2×	≥300	0.882	>5,514,392	[24]
*Acropora millepora*	1.5×	193	0.94	Unknow	[28]
*Chlamys farreri*	0.5×	174	0.91	3,968,417	[30]
*Haliotis discus hannai*	1×	≥400	0.98	147,449	the present study

## Data Availability

The data that support the findings of this study are available from the corresponding author upon reasonable request.

## Supplementary Materials

The supporting information can be downloaded at: https://www.mdpi.com/article/10.3390/ijms26104598/s1.
