# Supplementary material for: Evaluation of Low-Coverage Sequencing Strategies for Whole-Genome Imputation in Pacific Abalone Haliotis discus hannai
Source: Int J Mol Sci. 2025 May 11;26(10):4598. doi: 10.3390/ijms26104598 (PMC12111473; doi:10.3390/ijms26104598)
Supplement: Supplementary file 1 [file ijms-26-04598-s001.zip › ijms-3557757-supplementary.pdf]

Supplementary data：

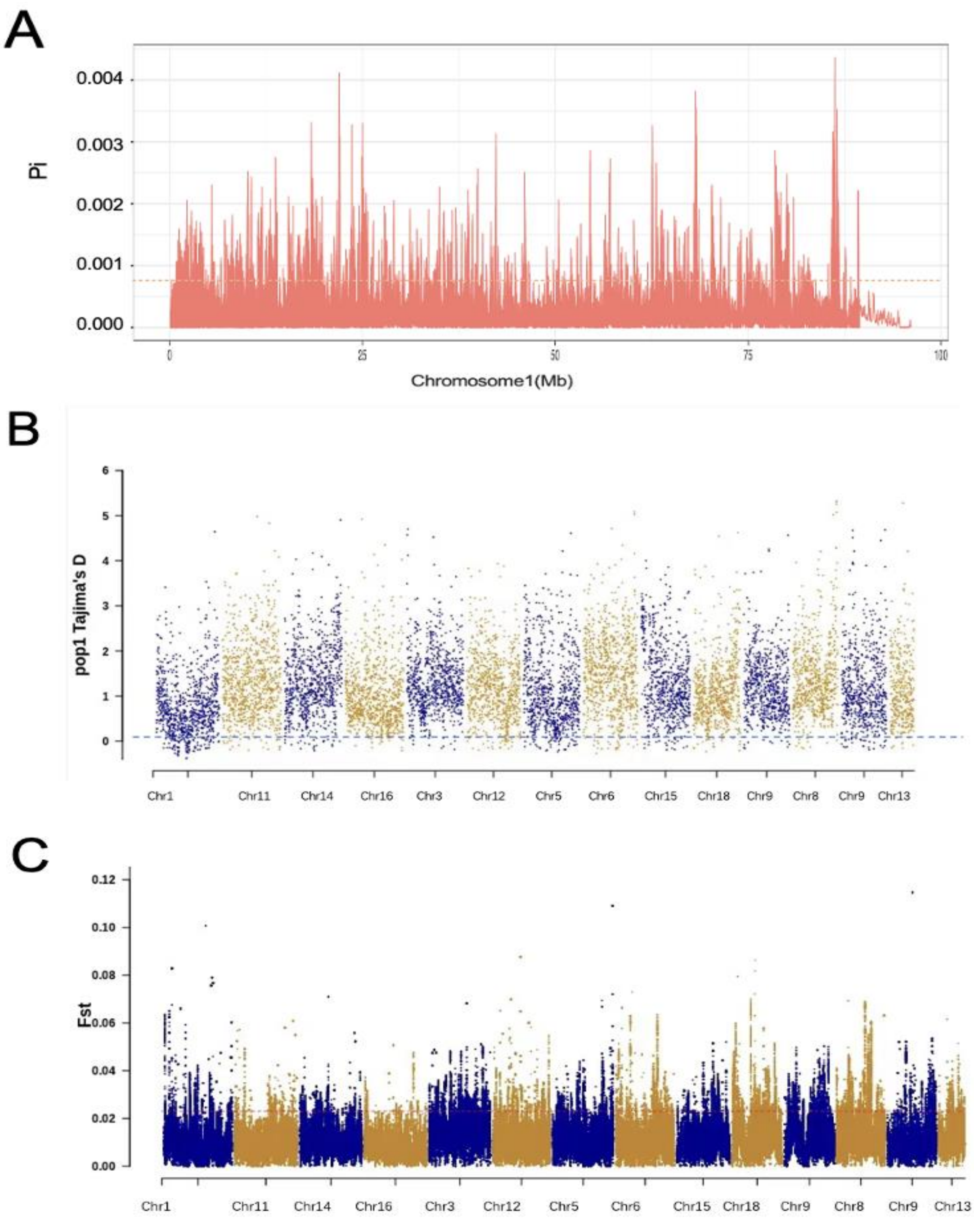

Figure S1. Population genetic structure of the 1,059 Pacific abalone samples with average sequencing depth of 7.86×. (A) Nucleotide diversity. (B) Tajima's D. (C) Fixation index
